# Smoking attitudes among medical personnel: A cross-sectional study at Mureș County Hospital, Romania

**DOI:** 10.18332/tpc/202966

**Published:** 2025-04-16

**Authors:** Corina Marginean, Bianca L. Grigorescu, Andreea C. Safta, Corina Budin, Nimrod Laszlo, Cristina A. Man, Septimiu T. Voidazan

**Affiliations:** 1 Mures County Clinical Hospital, Targu Mures, Romania; 2 Department of Oncology, Faculty of Medicine, George Emil Palade University of Medicine, Pharmacy, Science and Technology of Targu Mures, Targu Mures, Romania; 3 Department of Anesthesiology and Intensive Care, Faculty of Medicine, George Emil Palade University of Medicine, Pharmacy, Science and Technology of Targu Mures, Targu Mures, Romania; 4 Department of Pathophysiology, Faculty of Medicine, George Emil Palade University of Medicine, Pharmacy, Science and Technology of Targu Mures, Targu Mures, Romania; 5 Department of Pneumology, Faculty of Medicine, George Emil Palade University of Medicine, Pharmacy, Science and Technology of Targu Mures, Targu Mures, Romania; 6 Department of Biochemistry, Faculty of Medicine, George Emil Palade University of Medicine, Pharmacy, Science and Technology of Targu Mures, Targu Mures, Romania; 7 Department of Epidemiology, Faculty of Medicine, George Emil Palade University of Medicine, Pharmacy, Science and Technology of Targu Mures, Targu Mures, Romania

**Keywords:** smoking, hospital, medical staff, COVID-19, SARS-CoV-2

**Dear Editor,**

Smoking and other forms of nicotine addiction remain a major public health issue^1-4^. While global efforts to promote smoking cessation are ongoing, it is of interest to understand healthcare professionals’ attitudes and behaviors toward smoking. Understanding these shifts is critical, as healthcare professionals play a key role in tobacco control and public health promotion^5^. We aimed to fill this gap by examining how perceptions and attitudes toward smoking have changed before and after the COVID-19 pandemic among healthcare professionals at Mureș County Clinical Hospital, Romania.

A cross-sectional study, approved by the Ethics Committee of Mureș County Clinical Hospital (no. 8479/29.05.2023), was conducted from October to December 2023. Out of 326 hospital employees, 142 (43.9%) completed a questionnaire assessing their knowledge about smoking, changes in smoking behavior due to the pandemic, and attitudes toward smoking. The primary outcomes included post-pandemic smoking cessation or initiation, as well as changes in smoking frequency. Data were analyzed using IBM SPSS, with statistical significance set at p<0.05.

Regarding the perception of smoking’s impact on personal health, younger healthcare professionals (age 20–29 years) showed lower awareness of smoking risks (69.2%) compared to those aged 30–49 years (87.9%, p=0.020). Women demonstrated greater awareness of the risks (77.8%) than men (62.5%, p=0.027). Concerning changes in smoking behavior during the pandemic, most respondents maintained their smoking habits, with no significant differences between age groups. However, more women reduced their smoking (37.1%) compared to men (31.2%), who largely continued their habits (p=0.049). Approximately 34.5% believed smoking increased the risk of infection, and 52.1% felt it worsened disease severity. Significant differences by job role were identified (p=0.003), with doctors and residents being more aware of the risks of COVID-19 than auxiliary staff. Finally, a total of 82% expressed some intention to quit smoking, with no significant differences noted by age or gender (Figure 1).

**Figure 1:**
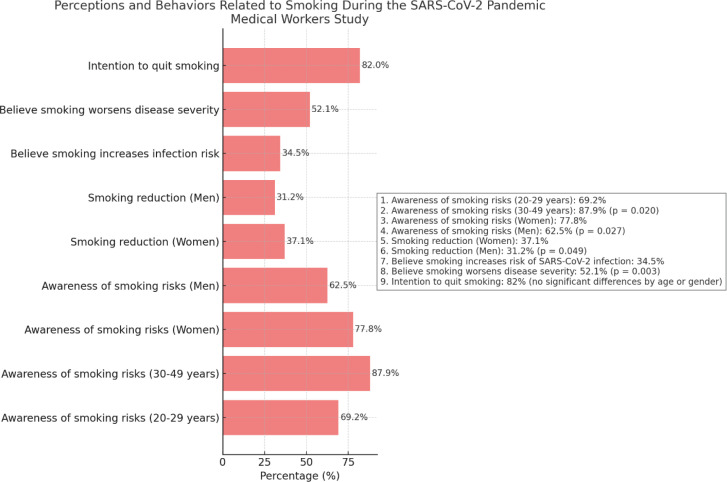
Smoking related attitudes among medical personnel, Mureș County Hospital, Romania 2023

These findings highlight the need for tailored educational campaigns to address the differing perceptions of smoking risks among healthcare workers in Romania. While the study’s cross-sectional design and limited sample size may affect the generalizability of the results, the insights gained are valuable for improving tobacco control measures at the regional level.

## Data Availability

The data supporting this research are available from the authors on reasonable request.
